# Stabilization of diastolic calcium signal via calcium pump regulation of complex local calcium releases and transient decay in a computational model of cardiac pacemaker cell with individual release channels

**DOI:** 10.1371/journal.pcbi.1005675

**Published:** 2017-08-08

**Authors:** Alexander V. Maltsev, Victor A. Maltsev, Michael D. Stern

**Affiliations:** Laboratory of Cardiovascular Science, Intramural Research Program, National Institute on Aging, NIH, 251 Bayview Blvd., Baltimore, MD, United States of America; University of California San Diego, UNITED STATES

## Abstract

Intracellular Local Ca releases (LCRs) from sarcoplasmic reticulum (SR) regulate cardiac pacemaker cell function by activation of electrogenic Na/Ca exchanger (NCX) during diastole. Prior studies demonstrated the existence of powerful compensatory mechanisms of LCR regulation via a complex local cross-talk of Ca pump, release and NCX. One major obstacle to study these mechanisms is that LCR exhibit complex Ca release propagation patterns (including merges and separations) that have not been characterized. Here we developed new terminology, classification, and computer algorithms for automatic detection of numerically simulated LCRs and examined LCR regulation by SR Ca pumping rate (Pup) that provides a major contribution to fight-or-flight response. In our simulations the faster SR Ca pumping accelerates action potential-induced Ca transient decay and quickly clears Ca under the cell membrane in diastole, preventing premature releases. Then the SR generates an earlier, more synchronized, and stronger diastolic LCR signal activating an earlier and larger inward NCX current. LCRs at higher Pup exhibit larger amplitudes and faster propagation with more collisions to each other. The LCRs overlap with Ca transient decay, causing an elevation of the average diastolic [Ca] nadir to ~200 nM (at Pup = 24 mM/s). Background Ca (in locations lacking LCRs) quickly decays to resting Ca levels (<100 nM) at high Pup, but remained elevated during slower decay at low Pup. Release propagation is facilitated at higher Pup by a larger LCR amplitude, whereas at low Pup by higher background Ca. While at low Pup LCRs show smaller amplitudes, their larger durations and sizes combined with longer transient decay stabilize integrals of diastolic Ca and NCX current signals. Thus, the local interplay of SR Ca pump and release channels regulates LCRs and Ca transient decay to insure fail-safe pacemaker cell operation within a wide range of rates.

## Author summary

Life’s vital rhythm, the heartbeat is guaranteed by specialized, cardiac pacemaker cells. Based upon the Hodgkin-Huxley membrane excitation theory and its application to cardiac cells, the cardiac pacemaker clock has been, for a long time, to be considered essentially a surface membrane oscillator, i.e. a membrane clock, driven by an ensemble of time- and voltage-dependent ion channels. More recent studies, however, discovered a tight integration of the membrane clock with an intracellular Ca oscillator (dubbed Ca clock). The Ca clock generates rhythmic, diastolic locally propagating Ca releases from sarcoplasmic reticulum, a cell major Ca store, that is refilled with Ca via a Ca pump. The released Ca activates Na/Ca exchanger that generates an inward current and accelerates the diastolic depolarization. Despite their importance, the local releases have not been systematically studied. Here we developed a new computer algorithm for automatic detection, classification, and analysis of local Ca releases generated by a recent computational model of a sinoatrial node cell. Then we investigated how the releases are regulated by the Ca pump, a major functional component of Ca clock. We discovered counterintuitive behaviors stabilizing diastolic Ca signal mass and ensuring fail-safe pacemaker operation at various pumping rates.

## Introduction

The Coupled Clock Theory [1] predicts an important role for the sarcoplasmic reticulum (SR) in the pacemaker function of the sinoatrial node cells. Changes in the SR Ca pumping rate (Pup) alone can regulate the pacemaker rate within the entire physiological range, including extremely low and high rates. The important functional role of Ca pump has been also suggested in experimental studies [2, 3] and in more recent numerical model simulations [4, 5].

This regulation is linked to the Local Ca releases (LCRs), which are SR discharges below the cell membrane [6, 7], and also to Ca transient decay [8]. The LCRs occur when SR gets refilled with Ca to a threshold level allowing spontaneous Ca release. The refiling time and the timing of the LCR occurrence are controlled by SERCA (SR Ca pump molecules) together with cell Ca available for pumping and intra-SR Ca diffusion. LCRs, in turn, accelerate the diastolic depolarization via activation of NCX current (review [9]).

The LCRs are generally larger in geometrical extent than isolated Ca sparks seen in ventricular myocytes, appear to be propagated locally, but do not form global waves. In simulations we generally use the term “spark” to refer to regenerative releases that appear to originate in a single couplon. While complex Ca releases also happen in ventricular myocytes (such as abortive waves or macrosparks, review [10]), they are not observed under normal physiological conditions. The release distinction is probably due to the underlying RyR distribution, which in sinoatrial node cells consists of irregularly placed sub-sarcolemmal clusters of various sizes (dubbed “hierarchical clustering” [5]) as opposed, for example, to sarcomeric intracellular arrays in ventricular myocytes. The local geometry of Ca diffusion probably also plays a role. Finally, in sinoatrial node cells, in contrast to ventricular myocytes, Ca cycling proteins, including RyRs and phospholamban (regulating SERCA), are phosphorylated in the basal state by PKA [11]. The functional consequence of the phosphorylation is higher rates of Ca pump and release resulting in occurrence of the diastolic LCRs [11] and their attendant diastolic NCX current [7, 12, 13], lacking in ventricular myocytes under normal conditions.

While Ca sparks and waves have been thoroughly characterized [10, 14], the LCRs in sinoatrial node cells have been neither unbiasedly detected, nor systematically classified and analyzed. The existing spark detection algorithms in confocal line-scanning images [14, 15] and in 2D recordings [15–18] are mainly tuned for stereotyped (simple) release events in ventricular myocytes (or in neurons [19]); and as such they cannot be applied to LCRs in sinoatrial node cells featuring complex release propagation patterns (including merges and separations) and multiple release peaks.

**Fig 1:**
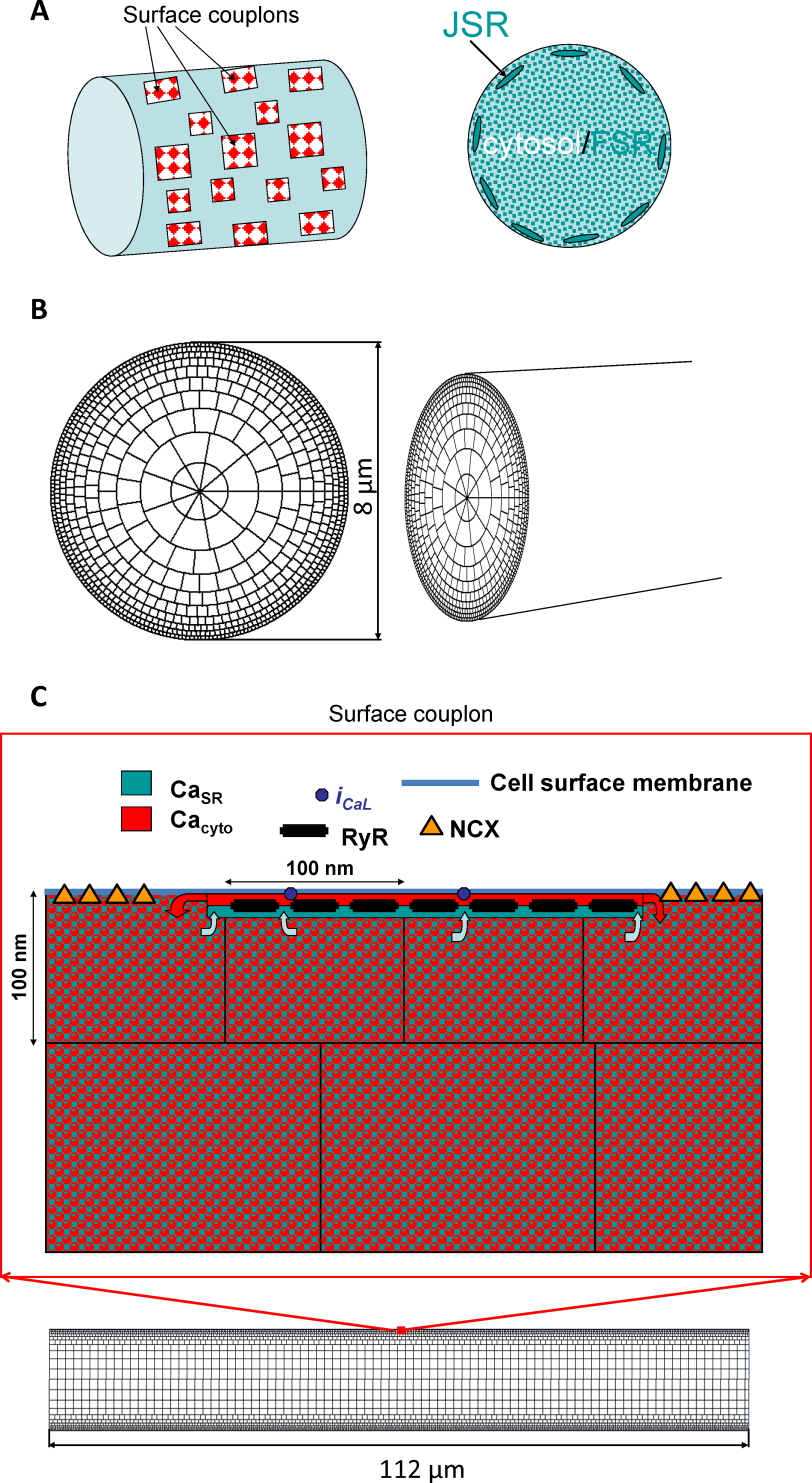
Schematic illustration of major components of numerical model of rabbit sinoatrial node cell used in our study. Abbreviations: Free SR (FSR) collects Ca from cytoplasm and transfers it to the junctional SR (JSR) that releases Ca under cell membrane; Ca_SR_, [Ca] in JSR; Ca_cyto_, [Ca] in cytoplasm; RyR, Ca release channel, ryanodine receptor; i_CaL_, L-type Ca channel; NCX, Na/Ca exchanger. See Methods for details. Modified from [5].

Thus, the aim of the present study was twofold:

1. to suggest a novel LCR classification and computer algorithm that automatically detects and analyzes LCRs generated by our recent model of sinoatrial node cells [5] (Fig 1).
2. to apply this new algorithm to explore LCR regulation by SR Ca pumping and its interplay with decaying Ca transient to form the integrated diastolic Ca signal activating NCX current.

We discovered counterintuitive behaviors of LCRs and Ca transient that stabilize the integrals of diastolic Ca and NCX signals upon variations in SR Ca pumping rate that insures regulation and fail-safe pacemaker cell operation within a wide range of action potential firing rates.

## Results

### LCR detection in a single frame

**Fig 2:**
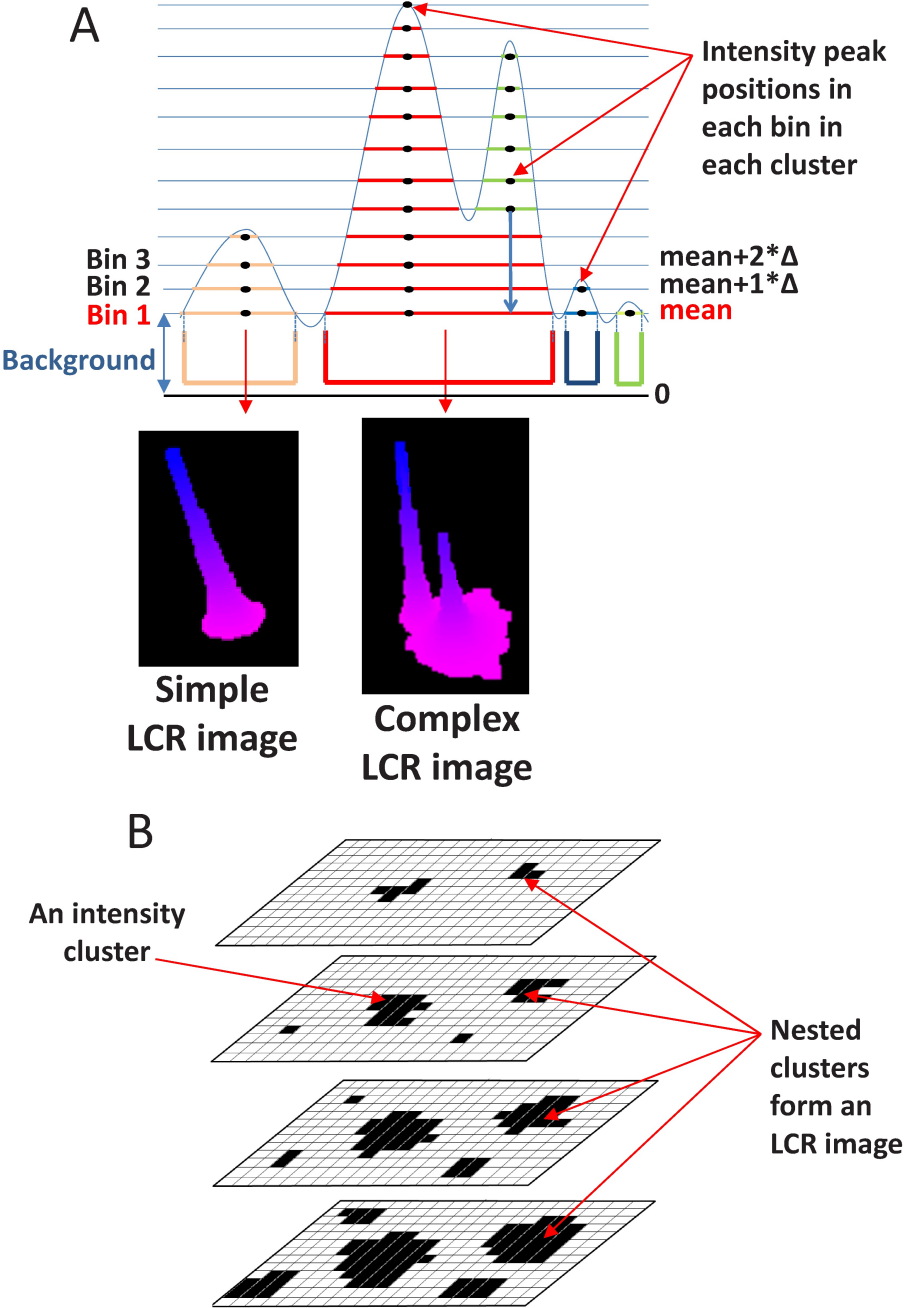
Detection of LCRs in a single frame (in all submembrane voxels at a given time). (A) A schematic one-dimensional representation of intensity binning of simple (left) and complex (right) release events. Intensity increment (Δ) is defined as (maximum [Ca]–mean [Ca])/50 and used to slice the frame into a set of intensity bins. Each such a bin is a Boolean array reflecting if [Ca] in a voxel > its binning level (horizontal blue lines), i.e. a multiple of the intensity increment above the mean. The lowest Bin 1 is illustrated with its binning level (set to mean value, red font). An intensity cluster in each bin are identified as a sub-set of the intensity bin whose TRUE Boolean elements have a common border. Intensity peak in each cluster is defined as [Ca] in a voxel that has the highest amplitude among its neighbors, so that a simple LCR image has only one intensity peak, but complex LCR image has multiple intensity peaks. Respective 3D examples of LCR images are shown in insets using OpenGL. (B). A schematic 2D representation of Boolean intensity binning to identify LCR images Black is TRUE while white is FALSE. Each LCR image is constructed as a part of a frame defined by the areas of nested clusters, i.e. all clusters sharing a lower bin.

In order to detect and characterize LCR in a given single time frame (reflecting an instant distribution of Ca) we introduced the definitions listed below. The definitions basically outline our LCR detection algorithm (see Fig 2 for more details).

**Frame**: an image (i.e. 2d-matrix) generated by numerical simulation program representing an instant [Ca] in each voxel directly under cell membrane.

**Intensity increment (**Δ**)**: an increment of [Ca] that is used to slice the frame into a set of intensity bins.

Δ=(highestamplitudeinframef–meaninframef)/(numberofbins).

**Intensity bin**: a mask (Boolean array) that reflects if [Ca] is higher of a given level defined by a multiple of the intensity increment over mean value. In our analysis the number of bins was 50 and the bins were constructed as follows:

(Bini,1<=i<=50)includedvoxelswith[Ca]>mean+Δ*(i-1)

The lowest intensity bin (Bin 1, Fig 2A) served the purpose of separating the LCRs from the background [Ca]. In other words, we considered all voxels with [Ca] above the average as a part of an LCR.

**Intensity cluster**: a sub-set of the **Intensity bin** whose TRUE Boolean elements have a common border (Fig 2B).

**LCR image**: a part of a frame within the area of the lowest bin of a set of nested clusters, i.e. all clusters sharing a common lower bin.

**Intensity peak**: [Ca] in a voxel that has the highest amplitude among its neighbors in a given intensity cluster.

**Simple LCR image**: an LCR image that has only one intensity peak

**Complex LCR image**: an LCR image that has multiple intensity peaks, i.e. all voxels with intensity peaks sharing a common low intensity bin within the LCR image.

**Signal mass (or total Ca) of LCR image (in nmol of Ca)**: a total [Ca] amount of an LCR image, calculated as a sum of Ca concentrations in nmol/liter in all voxels delineating the LCR image multiplied by the voxel volume given in liter (V_voxel_ = 100 nm*100 nm*100 nm = 10^−18^ liter).

$$\mathrm{Signal}\;\mathrm{Mas}s_{\mathrm{image}}=V_{voxel}\cdot \sum_{i=1}^{N}Ca_{i}$$

Thus, in a single frame, our computer program finds all LCR images. Each LCR image is described as a C++ object that represents a subset of submembrane cytosolic voxels, each of which features its own [Ca] and coordinates. Each LCR image object also carries the LCR signal mass (in nmol of Ca) and its complexity type (simple or complex). To handle all LCR images in a given frame, we create an array of objects of LCR images in that frame. Thus, each LCR image is fully referenced by its index within [1, the total number of LCR images] in that array and time (frame index) used further to describe LCR dynamics

### Algorithm for detection of LCR dynamics

**Fig 3:**
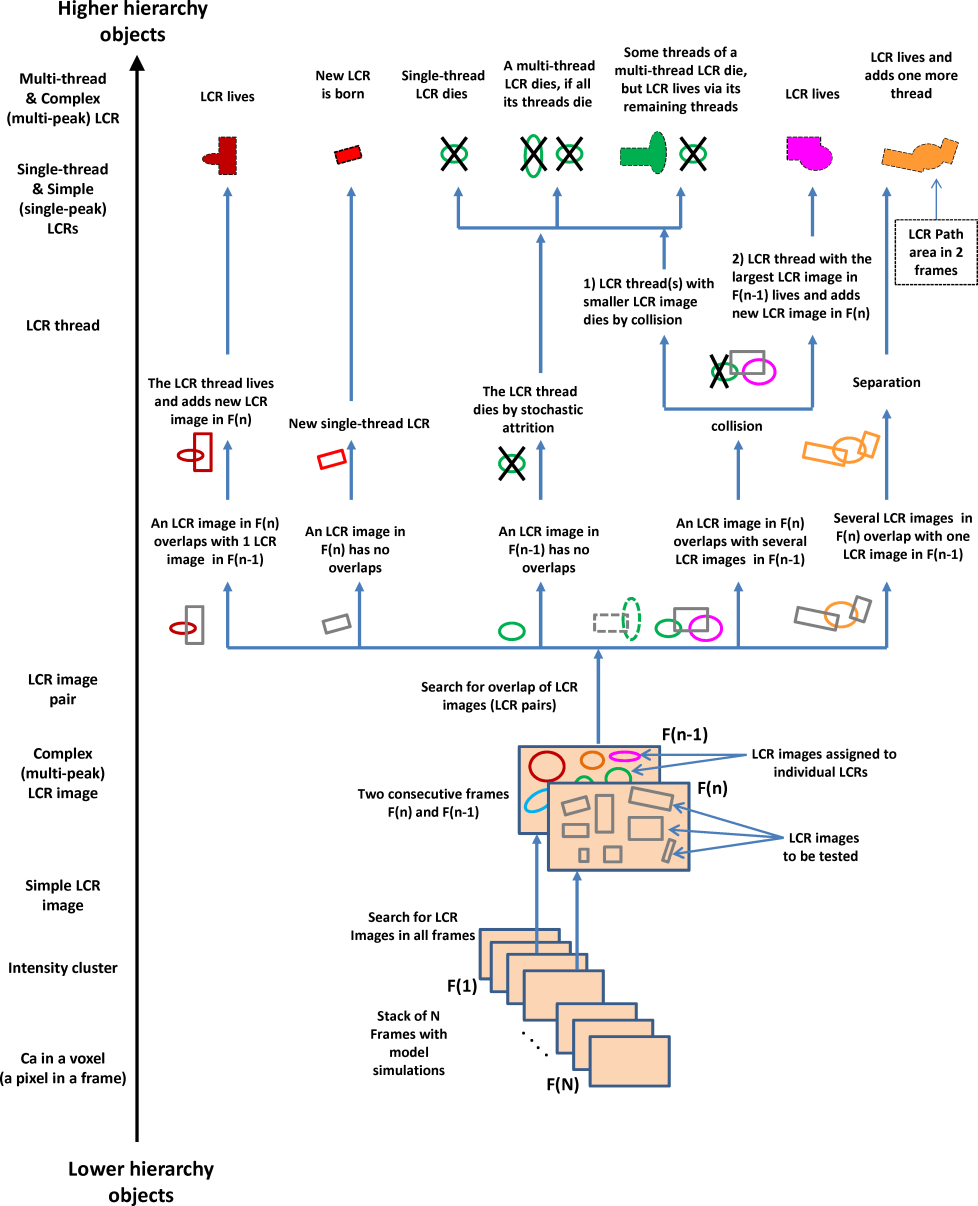
Schematic summary of our algorithm to detect and track LCRs. The algorithm is shown from bottom to top, as hierarchy and complexity of objects increase. The first step includes a low-level analysis of Ca distribution among neighboring voxels in each frame to construct intensity clusters and then LCR images. Next, the LCR images compared in consecutive frames to find overlapping pairs which are further used to track LCRs and their threads. The LCR images of previously identified LCRs in frame n-1 are illustrated by ovals of different colors. The LCR images to be tested in frame n are illustrated by grey squares. Different scenarios of LCR image overlaps are shown with respect to LCR birth, death, live, collision, and separations. The filled shapes in the top row illustrate LCR path area in the two consecutive frames n-1 and n. LCRs can exhibit different spatiotemporal complexity: they can split and then have several threads. A multi-thread LCR terminates only when all its threads terminate. LCRs can also include complex LCR images with multiple neighboring peaks, dubbed complex LCRs, and analyzed separately in our *in silico* investigation of SR Ca pump.

In order to identify, track, and classify LCRs in a series of time frames, we find all LCR images in each frame and then perform frame-to-frame comparison. Our complete analysis is schematically illustrated in Fig 3 from bottom to top, as hierarchy and complexity of objects increase.

The LCR represents the highest hierarchy object in our analysis and the algorithm uses the following definitions:

**Dynamic LCR (or simply LCR)**: a set of related LCR images describing in time and space either an individual release or a collection of interacting local releases. In our computer program we describe each LCR by a C++ object holding indexes of all its LCR images in each frame and its termination type.

**Complex LCR**: an LCR that has at least one complex LCR image.

**Simple LCR**: an LCR that has only simple LCR images

**LCR image pair**: A pair of LCR images that share at least one common voxel of the lowest intensity bin in subsequent frames (i.e. in the previous and in the current frame). We identify all pairs to detect LCR propagation, collisions and separations.

**LCR collision**: LCRs collide when the current frame has an LCR image that forms a pair with at least two of the LCR images in the previous frame

**LCR separation**: an LCR undergoes separation when at least two of its LCR images in the current frame form a pair with one common LCR image in the previous frame. Important: when an LCR undergoes separation, it is still considered as the same LCR, i.e. all subsequent coupled LCR images remain within the same C++ object tracking the LCR.

**LCR thread**: a subset of LCR images within a given LCR that represents a separate part of the LCR after its separation.

**Multi-thread LCR**: an LCR that has a history of separations.

**Simple dynamics LCR**: a single-thread LCR that has no history of separations.

**LCR max amplitude**: Maximum [Ca] that is reached by the LCR during its lifetime in μM.

**LCR path area (in nm**^**2**^**):** the total area that include all voxels participating in the LCR during its lifetime.

Signal mass of an LCR (in nmol*ms): the sum of signal masses of all LCR images constituting the given LCR multiplied by the time increment (5 ms).

### Types of LCR death (or termination):

1. *LCR is terminated by stochastic attrition*, when it has no overlap with any LCR images in the next frame.
2. *LCR is terminated by collision*, when the next frame has an LCR image that shares common voxels with the given LCR and another LCR that has a larger signal mass.
3. *LCR is terminated by Ca transient*, when an LCR is still alive on the last frame of the diastole, just before the action potential-induced Ca transient starts. Note: this termination type did not happen in our analysis, because we studied LCRs during diastolic depolarization below -50 mV, i.e. below activation of L-type Ca current (I_CaL_) and, therefore, before action potential-induced Ca transient.

### Tracking Simple LCR Dynamics

**Fig 4:**
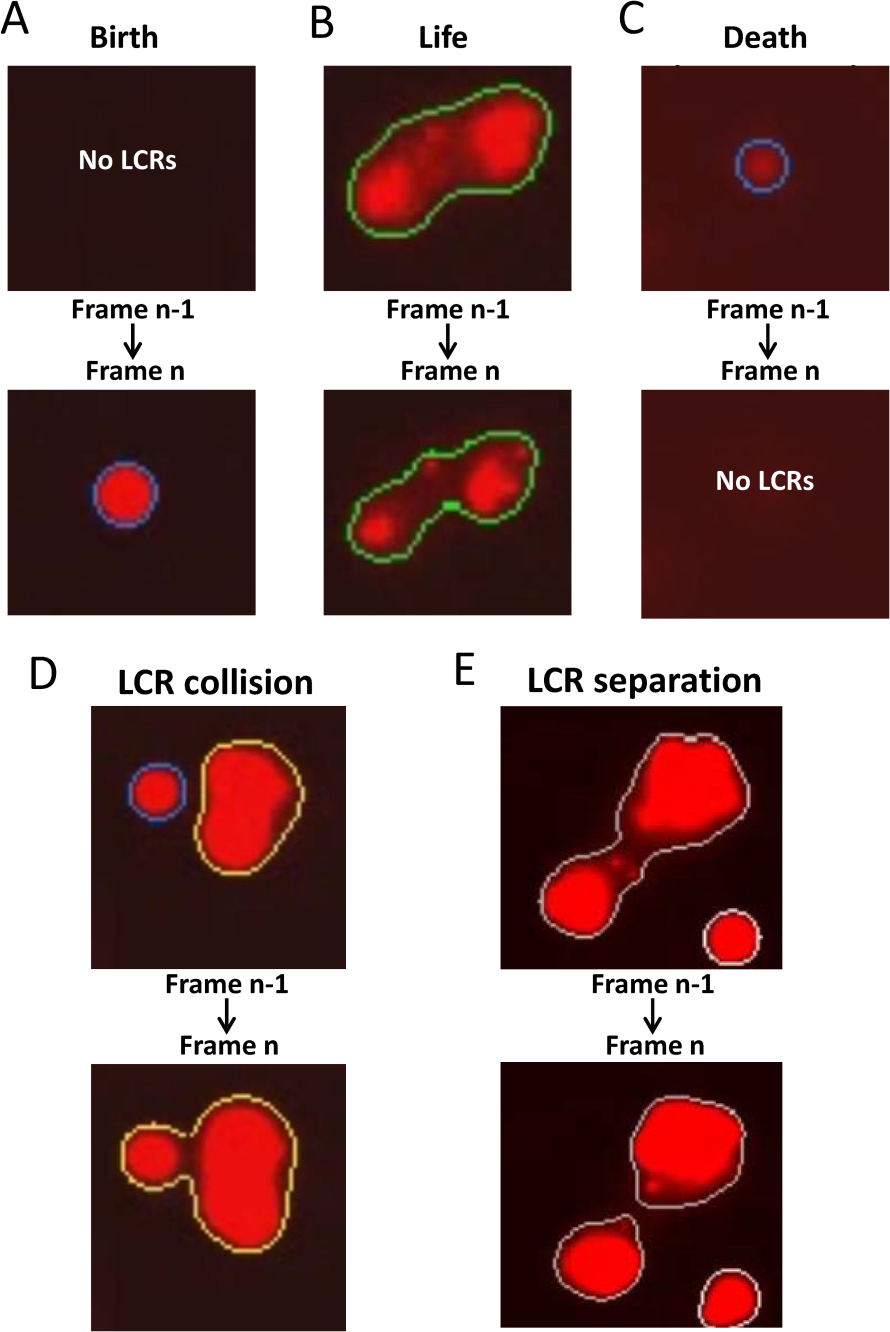
Examples of key LCR events. (A) Birth: When a LCR image from the current frame has no partner from the previous frame, then this designates the birth of a new LCR. (B) Life: In simple LCR dynamics LCR pair has a unique LCR image from the previous frame and a unique LCR image from the current frame. (C) Death: When an LCR image from the previous frame has no partner in the current frame, then the LCR that includes that LCR image is terminated by stochastic attrition. (D) Collision of two single-thread LCRs: LCR image pairs have the same new LCR image. The smaller LCR is pronounced dead by collision. The larger LCR continues to live. (B) Separation of a single-thread LCR into a two-thread LCR. The larger LCR in frame n-1 has two threads after the separation in frame n.

We define an LCR as a time-sequenced collection of LCR images. Each LCR has its birth with an initial image and then it adds more images to its collection as it progresses through frames. In the course of an LCR’s life, its ordinary behaviors include propagation, expansion, and/or contraction. A frame-to-frame comparison of LCR images is conducted to understand which LCR images in the current frame relate to the ones in the previous frame (Fig 4). Our algorithm finds all overlapping (i.e. by their spatial extent) LCR images in the previous frame and the current frame. Specifically, a Boolean (AND) matchup is made within the lowest intensity bins (outskirt) for all LCRs images found in the two frames. Thus our computer program creates a set of all LCR image pairs from the previous to current frame.

In simple LCR dynamics, an LCR image from the previous frame overlaps with a unique LCR image from the current frame (Fig 4B). The matched-up LCR image from the current frame is added to the LCR that its partner belongs to. Thus, the LCR is “built” and progressing as the algorithm goes through the frames (technically, the LCR object simply collects indexes of its linked LCR images). However this is not always the case. Sometimes a pair does not have a unique LCR image from the previous frame or from the current frame, reflecting collisions or separations (described later in the text).

In the case when an LCR image has no overlap with any LCR image from previous frame (Fig 4A), the algorithm initiates a new LCR (i.e. an LCR birth). In the case when none of the LCR images of an LCR in the previous frame finds a partner in the current frame (Fig 4C), the LCR is pronounced terminated by stochastic attrition (dubbed as LCR “death”).

### Definition of simple and complex LCRs

Next step is to identify whether a terminated LCR is complex or simple. If the LCR has at least one complex LCR image, then it is defined as complex. If an LCR consists of only simple LCR images, then the LCR is defined as simple. Because complex LCRs embrace several release sites, they usually exhibit complex dynamic behavior and last a long time, while simple LCRs (usually from one release site) last a short time. If the LCR persists until the very last frame, it is not a given that the LCR terminates, thus the LCR is labeled “still alive.” At the end of frame-to-frame comparisons, the total signal mass of each LCR is calculated by summing the signal masses of all its constituent LCR images and then multiplying by the time interval between frames (that is 5 ms).

### Exceptions to ordinary dynamics (collisions and separations). Multi-thread LCRs

With propagation in play, an LCR image from the current frame can form a pair with multiple LCR images in the previous one and vice versa, i.e. an LCR image from the previous frame can form a pair with multiple LCR images in the current one (Fig 3). These cases translate to either a collision or separation between LCRs, respectively. There are even cases where separation and collision occur simultaneously.

In the event of a collision (Fig 4D), we compare the signal masses of the previous LCR images of each pair, and the one with the largest signal mass has its pair’s LCR live on, while the rest have their LCR terminated, pronounced terminated by collision. The terminated LCR image pairs are also deleted from the main set of LCR image pairs, so that if LCRs collide and separate at the same time, the collision algorithm forcefully has priority over the separation algorithm.

In an event of a separation (Fig 4E), all the current LCR images from all the overlapping LCR image pairs are added to the LCR that the common previous LCR image belongs to. With separations, an LCR could be described in a space-time graph as a tree with multiple branches. Because branching happens in time we call the branches LCR “threads”. Thus, in this terminology, an LCR with a history of splits is a multi-thread LCR, whereas an LCR with simple dynamics (described above) can be described as a single-thread LCR.

As a clarification to a statement made in the previous section on LCR stochastic attrition, termination of multi-thread LCRs differs from that of single-thread LCRs. A multi-thread LCR is pronounced dead only when all its threads terminate (Fig 3, top). For example, if only one of the threads terminates, the LCR continue to live via remaining threads. In fact, even if all threads terminate, but one, the LCR still lives via the remaining thread.

### Investigation of the role of the SR Ca pumping in LCR statistics

**Fig 5:**
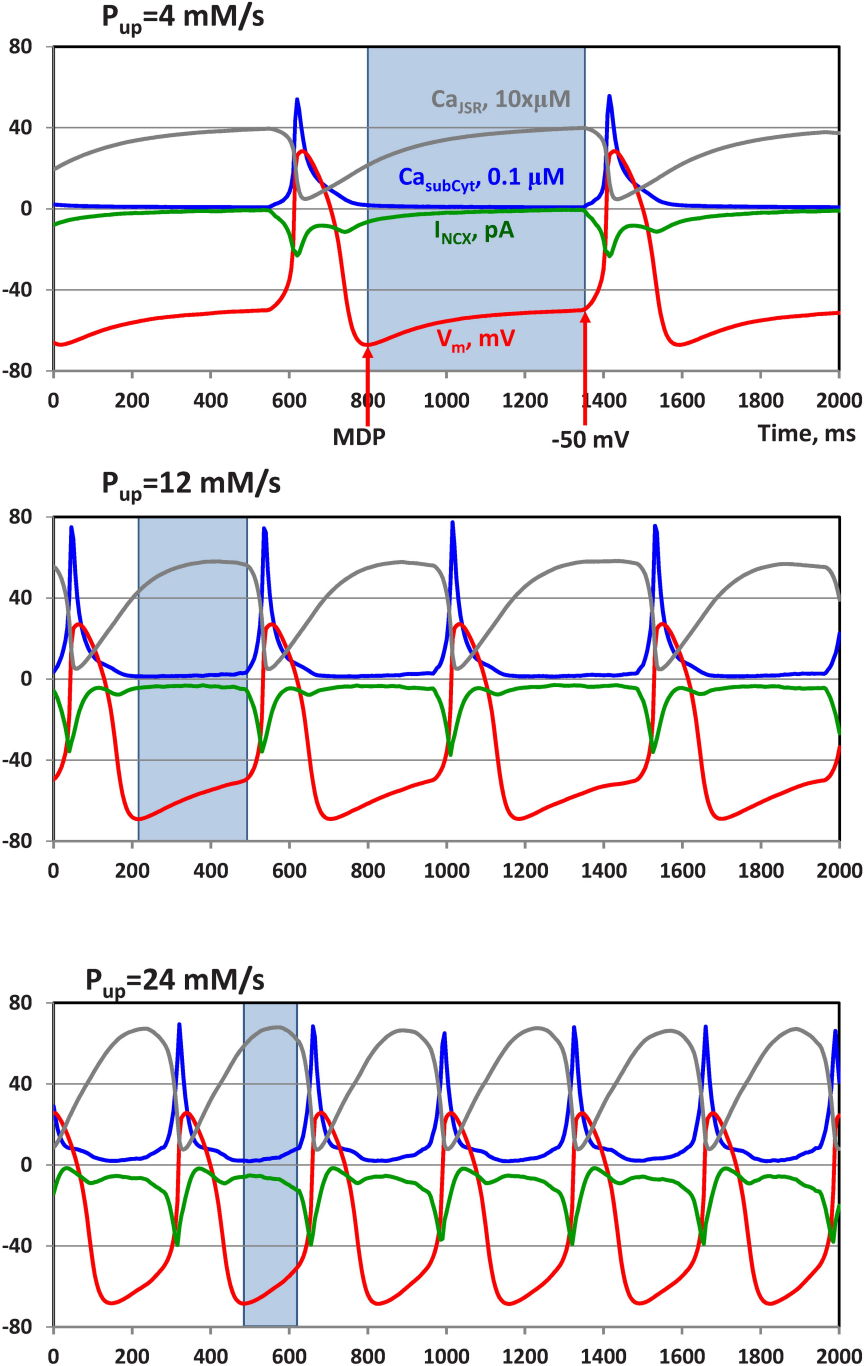
Increase in SR Ca pumping rate accelerates action potential firing rate in simulations of our numerical model of rabbit sinoatrial node cell. Shown are simulated traces of NCX current (I_NCX_, green traces), membrane potential V_m_ (red traces), and average Ca dynamics in submembrane layer of voxels (Ca_subCyt_, blue traces) and in junctional SR (Ca_JSR_, grey traces), at three values of Pup 4, 12, and 24 mM/s (shown above the respective panels).

We applied our algorithm of LCR detection and classification to get new insights into calcium pumping role in sinoatrial node cell function, specifically to identify the difference in statistics of LCRs generated by the SR at high (24 mM/s), moderate (12 mM/s), and low Pup (4 mM/s). At all Pup values, JSR (gray traces in Fig 5) becomes depleted of Ca by action potential-induced Ca transient (blue traces), but at higher Pup, JSR refilled quicker with Ca during diastole to reach a threshold of spontaneous Ca release. The earlier and stronger Ca releases activate stronger and earlier NCX currents (green traces), which shorten diastolic depolarization and the cycle length, so that the action potential firing rate increases (red traces) as predicted by the coupled-clock theory [1] (see also S1 and S2 Movies for more details, including local Ca dynamics).

**Fig 6:**
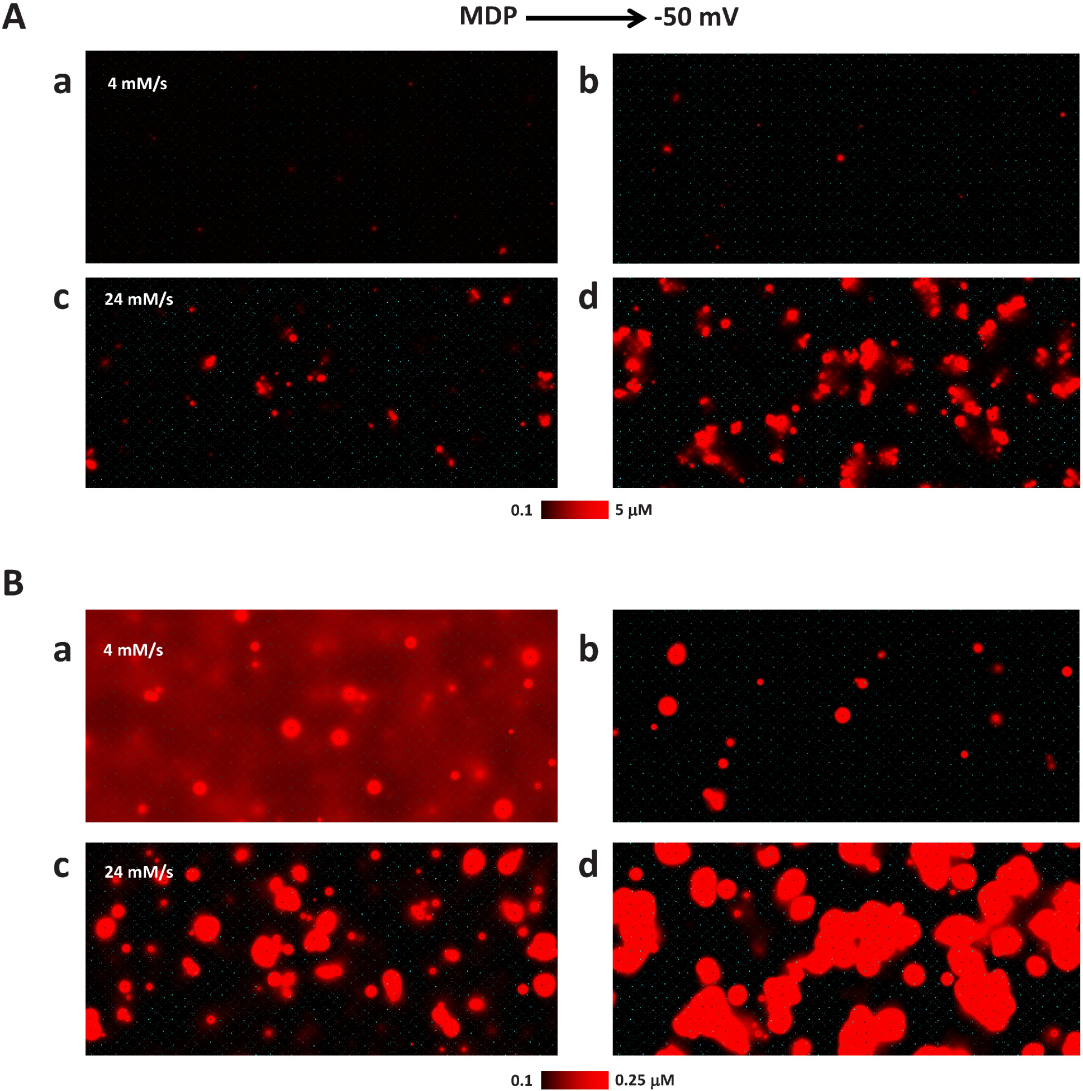
LCR activity and background Ca at low and high SR Ca pumping rate Pup of 4 mM/s and 24 mM/s as membrane depolarizes from the MDP (Maximal Diastolic Potential) to -50 mV (a threshold of I_CaL_ activation). (A): Local Ca distribution in submembrane cytosol voxels at the scale of 5 μM. The LCR activity increases during diastolic depolarization, but remains much lower at lower Pup. The background Ca does not appear different at this large Ca scale. (B) The same Ca distribution at a smaller scale of 0.25 μM. At low Pup, the cytosol is not efficiently cleared of Ca by the Ca pump after action potential-induced Ca transient and Ca distribution at the MDP (sub-panel a) shows substantially higher background and multiple small LCRs. Conversely, higher SR Ca pumping rate of 24 mM/s efficiently clears cytosol of Ca and the background Ca decreases (black background in sub-panel c). See also S1 and S2 Movies for more details and dynamic representation of the numerical model simulation results.

We examined Ca signals within a time window of the Maximal Diastolic Potential (MDP) to -50 mV, i.e. before the activation of L-type calcium current (the windows at various Pup are shown by a blue box in Fig 5). The MDP values were as follows: -67.2, -69.1, -68.5 mV for Pup 4, 12, and 24, respectively. The LCR activity generally increases within this time window. However, LCRs appear rare and small at Pup 4 mM/s, but large, frequent, and propagating at Pup 24 mM/s (Fig 6A). However, at the higher amplitude resolution of 0.25 μM local Ca distribution at low Pup shows substantially higher background Ca and multiple small LCRs at the MDP (Fig 6Ba vs. 6Bb).

**Fig 7:**
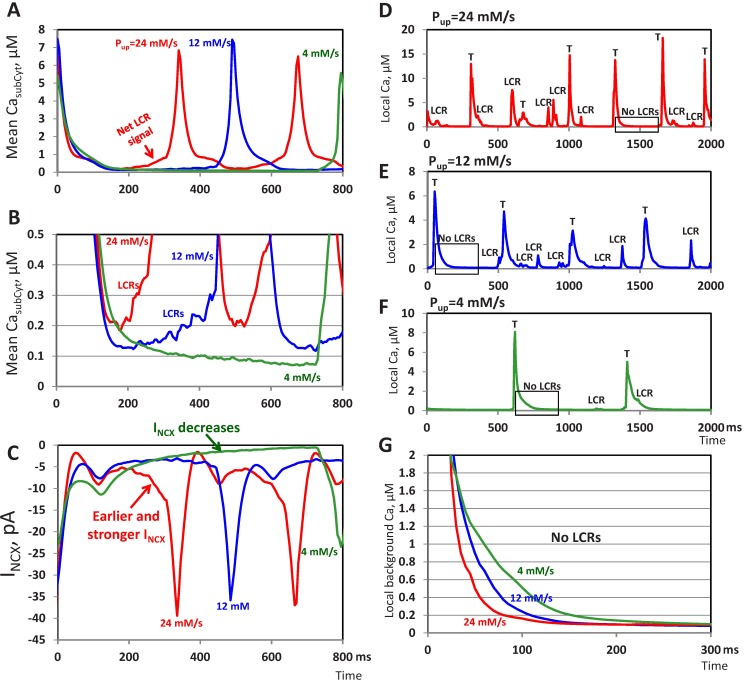
Analysis of average and local Ca signals in submembrane cytosol voxel to generate NCX currents during spontaneous action potential firing by our numerical model of sinoatrial node cell at various SR Ca pumping rate (Pup). (A and B) Overlapped average Ca signals synchronized at the Ca transient peak at different scales of 8 and 0.5 μM, respectively. The noisy rising Ca signal caused by LCR activity is marked by label “LCRs” in panel B. (C) The corresponding NCX currents simulated concurrently with the Ca signals in panels A and B. Occurrence of an earlier and stronger inward NCX current at high Pup is shown by red arrow. Green arrow shows decreasing NCX current at low Pup. (D-F) Examples of noisy local Ca dynamics in a single voxel at various Pup. LCR signals generated nearby are labeled “LCR” and the local Ca transient is marked “T”. Parts of Ca local transient decays with no LCR are outlined by rectangles. (G) Overlapped traces of the local Ca transient decays outlined in panels D-F. The decay is faster at higher Pup.

We further examined the interplay of LCRs and Ca transient decay (seen as decreasing Ca background) with respect to generation of the diastolic NCX currents important for diastolic depolarization and ultimately action potential firing rate (Fig 7). Overlapped Ca signals (synchronized at the Ca transient peak) show clear diastolic Ca elevations (Fig 7A) linked to LCR activity that increases at higher Pup (arrow “net LCR signal”). More important details can be seen at a smaller scale (Fig 7B), i.e. the LCR signal overlaps with the background (Ca transient decay), resulting in a higher nadir (Ca minimum at about 200 nM) at Pup = 24 mM/s vs. below 100 nM at Pup = 4 mM/s. The corresponding simulated NCX current traces show an earlier and stronger increase at 24 mM/s, but a decrease at 4 mM/s (Fig 7C).

To provide further insights into local Ca signaling, we illustrate typical Ca dynamics in a single voxel at various Pup (Fig 7D–7F). The dynamics is rather noisy at higher Pup, reflecting LCR occurrences nearby the voxel. To evaluate the background changes, we found parts of Ca local transient decays with no LCR occurrences nearby (shown by rectangles). Then we overlapped the traces to illustrate that indeed the decay is accelerated at higher Pup (Fig 7G), indicating that the higher nadir of total Ca signal (Fig 7B) is indeed due to LCR overlap, rather than a Ca overload.

**Fig 8:**
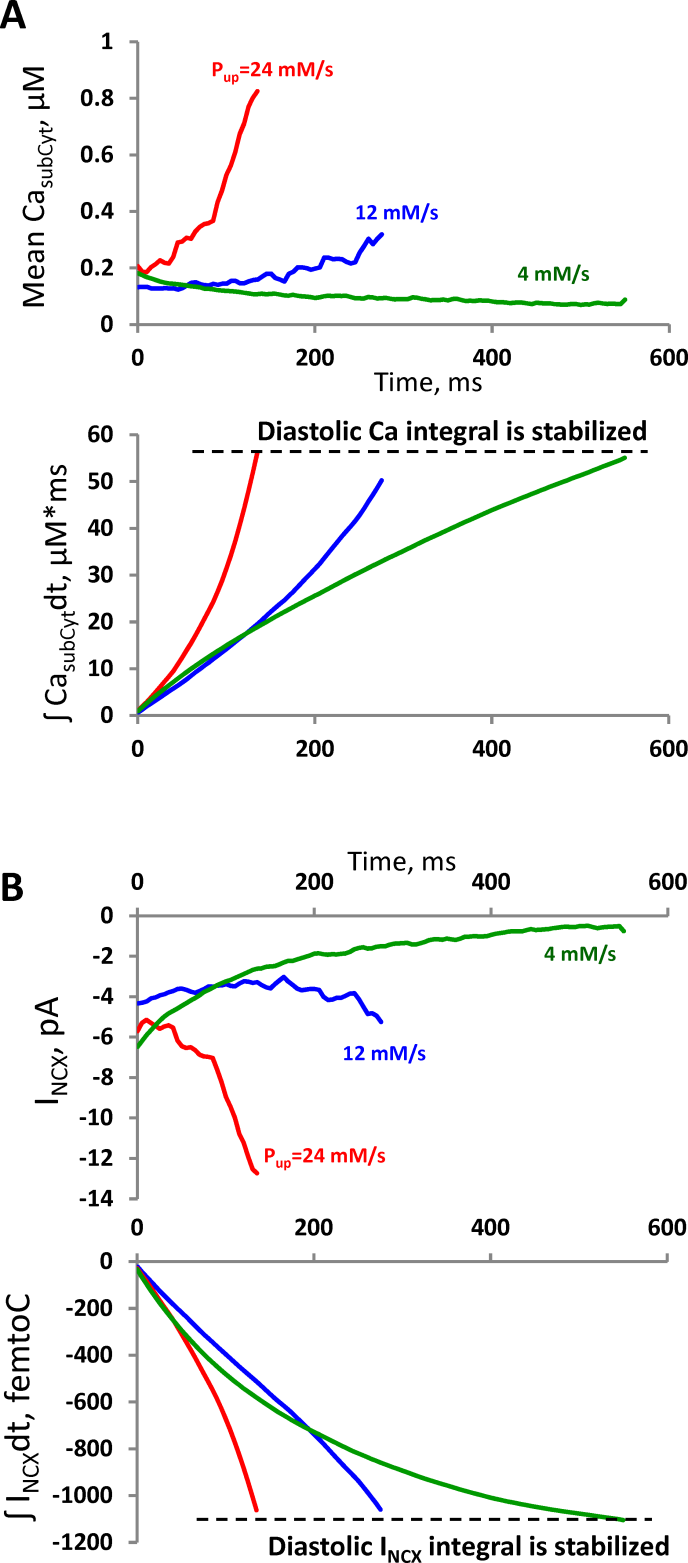
The integrals of diastolic Ca and NCX signals are invariants at various SR Ca pumping rate (Pup). (A) Overlapped average Ca signals (top) and their respective integrals (bottom) synchronized at the MDP and extended to the time when membrane potential reaches I_CaL_ threshold (-50 mV in our model). The Ca invariant is shown by a dash line “Diastolic Ca integral is stabilized”. (B) Respective NCX currents (I_NCX_) and their integrals. The NCX invariant is shown by a dash line “Diastolic I_NCX_ integral is stabilized”.

We next examined the net (integrated) Ca and NCX signals generated by the transient and LCRs in the time window of our interest from MDP to -50mV (Fig 8). Surprisingly, both Ca signal and NCX signal integrated over the critical time window during diastolic depolarization remained basically invariant, despite huge variations in Pup and action potential firing rates.

Next, we performed statistical analysis and comparisons of automatically detected LCRs in the same time window of interest (from MDP to -50 mV) at two extreme Pup of 4 and 24 mM/s. The analysis confirmed generation of numerous LCRs observed at the low Pup in S1 Movie and Fig 6B: in fact, nearly four times as many LCRs that appeared at higher Pup (Table 1). When we show LCRs spatial dynamics (without Ca amplitude grading) in S3 Movie, one can indeed observe frequent and large spreading LCRs at Pup 4 mM/s. As we show below, the majority of these LCRs is of relatively low amplitude and have a small impact on the net diastolic Ca signal.

**Table 1:** Key LCR characteristics measured at three SR Ca pumping rates Pup in each representative cycle.

Pup, mM/s	All LCRs	Complex LCRs	Simple LCRs
	**Total LCRs in Sample Cycle**		
24	443	195	248
12	551	243	308
4	1978	210	1768
	**Total Signal Mass (nmol×ms×10**^**−10**^**)**		
24	101	99.8	1.45
12	64.3	61.8	2.50
4	30.7	25.8	4.90
	**Signal Mass per LCR (nmol×ms×10**^**−10**^**)**		
24	0.229	0.512	0.00586
12	0.117	0.254	0.00813
4	0.0155	0.123	0.00277
	**Average Path Area (μm²)**		
24	3.9037	8.1171	0.5871
12	4.4113	8.3388	1.3127
4	2.2722	15.2278	0.7269
	**Average LCR Maximum Amplitude (μM)**		
24	24.7504	39.5754	13.0809
12	19.4819	30.2397	10.9944
4	3.0111	15.4352	1.5291
	**Average Duration (ms)**		
24	26.36	42.86	6.16
12	30.64	50.06	15.32
4	20.97	86.07	13.21
	**Average Birth Time (ms)**		
24	56.02	51.73	59.40
12	115.38	126.69	106.46
4	107.97	220.52	94.55
		**Total Complex Collisions**	
24		29	
12		24	
4		9	

**Fig 9:**
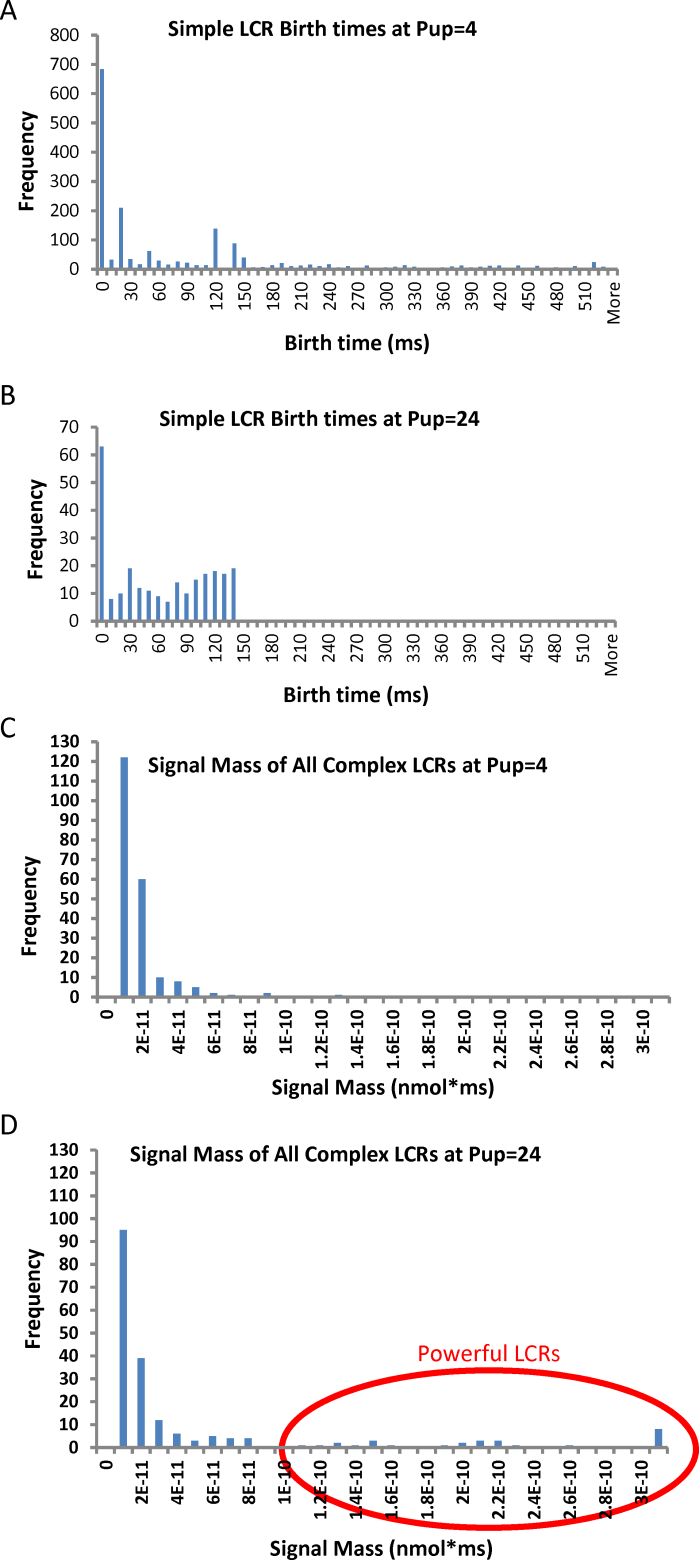
Initial statistical analysis of automatically detected LCRs. (A and B): Distributions of simple LCR birth times at different SR Ca pumping rates of Pup 4 mM/s and Pup 24 mM/s. Simple LCRs at Pup 4 mM/s appear mainly at the beginning of the diastolic depolarization period. The 0 bin takes all pre-existing LCRs as new and thus should not be taken into consideration. (C and D): Distributions of complex LCR signal mass at different Pup of 24 mM/s (C) and 4 mM/s (D). Powerful complex LCRs emerge at higher Pup (circled in red).

Next we applied our new LCR classification and found that the amount of complex LCRs stayed nearly the same, whereas the amount of simple LCRs was about 7 times larger at low Pup (see Table 1). With regard to the diastolic depolarization time course, most of the simple LCRs at low Pup formed closer to the start of the diastolic depolarization i.e. the MDP (histograms in Fig 9A and 9B), whereas complex LCR appeared throughout the entire diastole (see S3 and S4 Movies). Complex LCRs had an average birth time counted from the MDP of 221 ms, whereas simple LCRs had an average birth time of only 95 ms. The very premature small simple LCRs are actually linked to the decaying transient, provoking releases from the junctional SR (JSR) not completely filled with Ca. Poor SR Ca pumping at Pup 4 mM/s clears cytosolic Ca less efficiently, leaving much higher background Ca levels as clearly seen in Fig 6B (red background vs. black background in sub-panels a vs. b) and local decays lacking LCRs in Fig 7G.

As followed from histograms in Fig 9A and 9B, such small isolated releases happen less frequently further in the diastole because the transient has been decayed and the cytosolic calcium is cleared (Fig 7G), preventing premature releases and thereby allowing JSR to refill with substantial amount of Ca before the release. It is important to note, however, the early simple LCRs were much smaller and contributed much less toward the total diastolic signal vs. complex LCRs. Specifically, signal mass of all complex LCRs was 5 times larger than that of all simple LCRs at Pup 4 mM/s and 68 times larger at Pup of 24 mM/s (see Table 1). Taking into account this result we have assumed simple LCRs as negligible and focused our further analysis on complex LCRs.

While the number of complex LCRs are almost the same at both Pup, at high Pup complex LCRs had larger signal mass than those at low Pup. (Table 1, parameter “Signal Mass per LCR”). Our histogram of the LCR signal mass distribution revealed an emergence of high signal mass LCRs (Fig 9D, shown by red circle) that are not observed at low Pup (Fig 9C). We dubbed these release events “powerful LCRs”, because LCRs with higher signal mass (i.e. larger size and amplitude) is a known powerful mechanism to generate larger NCX currents and notably accelerate the pacemaker rate (e.g. in numerical simulations [12] and in experiments with phosphodiesterase inhibition [13]).

**Fig 10:**
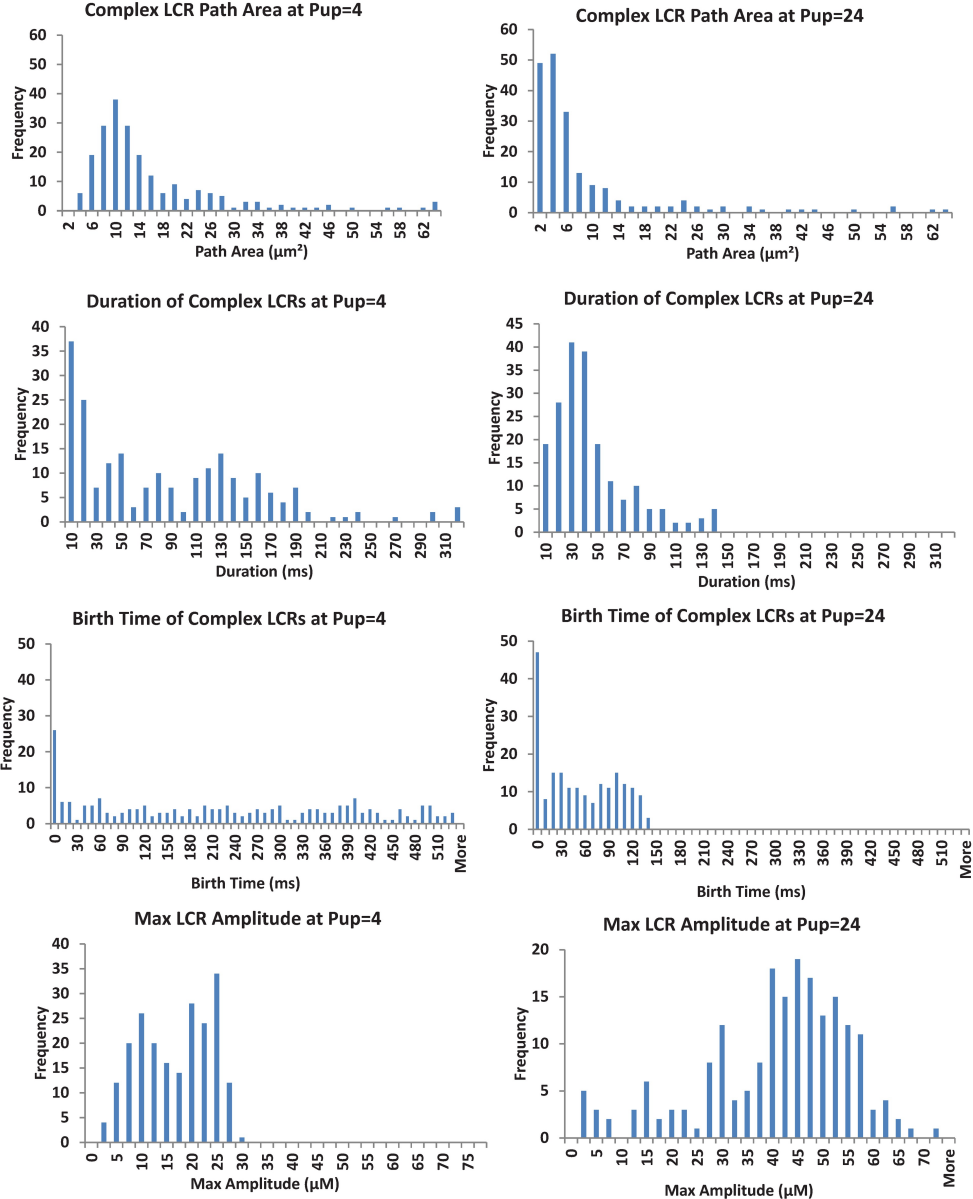
A detailed statistical analysis of key parameters of complex LCRs at different SR Ca pumping rates: Pup 4 mM/s (left) and Pup 24 mM/s (right). The LCRs at high Pup were not only more powerful and faster propagating, but importantly they occurred earlier in the diastolic depolarization and lasted shorter (see birth time and duration in Table 1), resulting in a more synchronized and higher amplitude LCR ensemble signal.

To get further insights into emergence of the powerful LCRs, we analyzed the components of the signal mass parameter: path area, duration, birth time, and amplitude. At Pup 4 mM/s the average LCR path area was larger and their duration was longer than that of Pup 24 mM/s (Table 1, “Average Path Area” and “Average Duration,” Fig 10). Thus, the major factor of a larger signal mass at Pup 24 mM/s was actually larger release amplitude (Table 1, “Average LCR Maximum Amplitude”). Our analysis provided more interesting results. Even though LCRs lasted shorter and their path areas were smaller at Pup 24 mM/s, the amount of collisions (Table 1, “Total Complex Collisions”) was larger at the high Pup, indicating a more active recruiting process i.e. Ca-induced-Ca-release (CICR), resulting in a faster propagation.

Finally, to further support our findings, we performed additional LCR analysis at a moderate SR Ca pumping with Pup of 12 mM/s (i.e. between the two extreme values of 24 and 4 mM/s discussed above) that was assigned to a basal state action potential firing in prior numerical studies [1, 20]. We measured parameters of LCRs (see Table 1) detected by our algorithm under these conditions and illustrated LCR detection in S5 Movie. The appearance of the detected LCRs and their parameters values were found somewhere reasonably between those described above at the two extreme Pup of 4 and 24 mM/s. An important result of this additional analysis was that at all three Pup tested the number of complex LCRs remained almost unchanged, and the total signal mass of all LCRs was composed of mainly complex LCRs. Based on data in Table 1, complex LCRs contributed 84%, 96% and 99% to the total signal mass of all LCRs at Pup 4, 12 and 24 mM/s, respectively.

## Discussion

The present study accomplished two goals: 1) we developed a novel LCR classification and computer algorithm that automatically detects and analyzes LCRs generated *in silico* [5]; 2) using the new algorithm we discovered a new mechanism of stabilization of integrals of diastolic Ca and NCX signals via LCR adoptive changes and an interplay of the LCRs and Ca transient over a wide range of SR Ca pumping rates.

Our new classification of LCRs includes LCR merges and separations as well as three types of termination (by stochastic attrition, merging into a larger release, and fusion into action-potential-induced transient). Using our new algorithms and our new terminology, we were able for the first time to classify (and thus simplify) the extremely complex appearance of intracellular Ca dynamics (S1 and S2 Movies) by a few typical cases of LCR behavior with their statistics and contributions into the total Ca signal.

We found that signal mass of all LCRs is mainly generated by complex LCRs over a wide range of pumping rates, indicating the functional importance of CICR (and release interactions) in forming the LCR signal and pacemaker cell function. Simple (non-propagating), small LCRs mainly occur close to the MDP at the end of the decaying transient. The incomplete transient decay, especially at low pumping rates at the beginning of diastolic depolarization (Fig 7G), results in a higher cytosolic Ca that provokes the premature small releases which have a small impact to the total LCR signal. Small LCRs at the beginning of the diastolic depolarization were also observed in 2D high-speed camera recordings in rabbit sinoatrial node cells [21].

Our examination of complex LCRs revealed a larger path area and longer duration for the LCRs at low Pup (Table 1). This counterintuitive result is explained by the fact that poor SR Ca pumping imparts a higher background diastolic Ca in the cytosol (Fig 7G) that provokes release propagation and therefore longer duration of the complex LCRs, despite their smaller amplitude. Furthermore, while the total LCR signal mass decreases at low Pup (Table 1) the duration of the diastolic depolarization becomes longer and slower Ca transient decay contribution to net NCX signal increases. This interplay of LCRs and the transient stabilizes the integrals of diastolic Ca and NCX signals (Fig 8).

This finding offers a new explanation to a prior experimental result that a substantial pharmacological inhibition of SR Ca pumping (e.g. by cyclopiazonic acid [2]) decreases the rate, but does not cease spontaneous action potential firing of sinoatrial node cells. This experimental result is often used as a key argument against importance of local Ca releases in cardiac pacemaker function (e.g. section “Are Ca^2+^ sparks/LCRs Necessary for Pacemaking?” in [22]). The present study shows that Ca releases are important to stabilize the Ca signal mass over a wide range of Pup, but especially at higher Pup. A similar counterintuitive result has been reported in NCX-deficient model, in which diastolic NCX current (and basal pacemaker rate) remained almost unchanged (i.e. stabilized), with only 20% remaining NCX molecules [23].

Next we found that the increase in the action potential firing rate at higher Pup is associated with a more synchronized and higher amplitude LCR ensemble signal that ultimately generated a higher amplitude NCX current, accelerating the diastolic depolarization (Ca_subCyt_, I_NCX_ and V_m_ traces in S1 and S2 Movies, Fig 7A–7C). That is in line with the coupled-clock theory ideas [1, 9] and prior experimental observations that at higher pumping rate (e.g. in the presence of β-adrenergic receptor stimulation) LCRs increase in amplitude, signal mass and emerge earlier within the cycle [11, 24]. On the contrary, in the presence of pharmacological inhibition of SR Ca pumping with cyclopiazonic acid, LCRs decrease in amplitude and signal mass and they emerge later within the cycle [3] that is also in line with our finds here in simulated LCRs.

Increase in the release amplitude (dubbed spark current or “I_spark_” in prior studies [25, 26]) is an important known mechanism of release recruitment via CICR [26]. Our statistical LCR analysis show increased number of LCR collisions and faster propagation at higher Pup that is an important mechanism of LCR synchronization. At the same time higher Pup depletes cytosolic Ca more efficiently, preventing propagation and premature releases. The preclusion of the premature small releases represents, in fact, yet another release synchronization mechanism. Finally, the faster SR Ca pumping is associated with faster SR refilling and more synchronous attainment of the spontaneous release threshold in different SR locations, representing the third release synchronization mechanism.

There is a discrepancy, however, between simulations and experiments with respect to both the LCR number and size when SR pumping varies. Partial inhibition of SR Ca pump with cyclopiazonic acid decreased the LCR number and size, but β-adrenergic receptor stimulation increased these parameters [3, 11, 24]. When we varied Pup *in silico*, the number of complex (meaningful) LCRs stayed almost unchanged (Table 1), but the LCR size changed to the opposite vs. the experimental results. This apparent contradiction could be explained by the fact that our algorithm detects all releases in the model system, in which there is no noise. Experimental records of fluorescent Ca indicator signals, however, have instrumental noise that precludes detection of the low amplitude releases (predicted by the theory). As LCR signal amplitude increases, the LCRs become experimentally detectable, observed as an apparent increase in LCR numbers (as seen in S1 and S2 Movies, in a high Ca scale of 5 μM).

There are seveal technical issues that complicate application of our LCR detection method to real pacemaker cells. Recording noise of high speed cameras and confocal microscopes is one of major issues. An additional issue is that experimental sub-sarcolemmal calcium is very difficult to visualize in real time due to dye bleaching, photo-damage, Ca buffering by indicator molecules, and scanning rate limitations, especially with 3D microscopy methods. Finally there is a motion artifact in these spontaneously contracting cells. that requires mapping the intracellular space via an additional algorithm.

Some of these issues have been explored in our recent pilot study, in which we applied the LCR detection principles and terminology reported here to experimental Ca recordings performed by high speed cameras in sinoatrial node cells isolated from rabbit and guinea pig [27]. The cell motion artefact was addressed by using non-Euclidean dynamic coordinate transformations to compensate for cell contraction. Similar to simulated LCRs analyzed here, LCRs in real cells also expand, propagate, merge, separate and terminate. However, the number of LCRs per cycle varied substantially among individual cycles, cells, species, and camera types, but rarely exceeded 100, indicating that many LCRs present in numerical simulations (Table 1) are missed in the real recordings. Some smaller release signals can be hidden within the experimental noise and remain below the detection threshold. In the absence of recording noise, our modeling system detects every LCR in curved submembrane space that is perfectly scaled in terms of space coordinates and absolute Ca concentrations, whereas the experimental data are obtained from noisy 2D non-confocal images of non-linear Ca sensing dyes, which are projections through the cell of various shapes (spindle shape, spider shape, etc.), convolved with the point-spread function of the microscope. Despite these numerous technical issues, our new algorithm and terminology seem to be helpful in statistical analysis and interpretation of the experimental data. Further technical advances in Ca signal recording will narrow the gap between the experimental and simulated data.

In summary, we introduced new terminology, classification, and automatic algorithms to characterize complex spatiotemporal structure of LCRs in cardiac pacemaker cells. Using this new approach, we found that Ca pumping regulates LCRs and pacemaker rate via timely synchronized occurrence of LCRs creating a powerful ensemble signal activating NCX current. Specific mechanisms of LCR synchronization include CICR, suppression of small premature releases, and more synchronous SR refilling with Ca at higher Pup. At lower Pup the LCRs have smaller amplitudes, but the system manifests a counterintuitive behavior to stabilize the diastolic Ca signal mass (and its NCX signal) by increasing LCR size and duration and by increasing the signal from slowly and longer decaying Ca transient. More generally, these results demonstrate functional importance of complex local crosstalk among NCX, LCR, Ca pump, and L-type Ca channels. The emergent behaviors of these interactions deserve accurate and more detailed future studies.

Finally, our development of new LCR detection and classification algorithms will be also helpful in creating similar approaches to unbiased detecting and analyzing experimental data on LCRs in sinoatrial node cells [27]. Specifically it would be important to compare LCR synchronization mechanisms *in silico* and experimental data via analysis of statistics of major LCR parameters introduced here, including path area, amplitude, duration, birth time, separations, collisions, and stochastic attritions. Highly integrated release events have also been reported in other cell types: Ca wavelets, abortive Ca waves, macrosparks, compound sparks or puffs [10]. Our new approach to detect and analyze complex local release events may be also helpful in studies of these Ca release events.

## Methods

### Numerical simulations

The numerical simulations were performed using our recent 3D-model of rabbit sinoatrial node cells [5]. The model structure is illustrated in Fig 1 and a full model description can be found in the original paper.

In short, membrane ion currents and voltage are represented by ordinary differential equations, except for the L-type calcium current, which is handled separately and stochastically. Diffusion and buffering of calcium in the cytosol, and in the free SR, are represented by partial differential reaction-diffusion equations. The free SR is treated as a fine random network, considered as a separate, continuous space co-extensive with the cytosol.

The model simulates the function of individual Ca release channels, ryanodine receptors (RyRs), located in a close proximity to the cell membrane in line with the prior experimental result that the primary pacemaker cells of smaller size in the center of rabbit sinoatrial sinoatrial node express RyRs mainly under cell membrane (along cell perimeter) with almost no presence in the bulky cytosol [5, 28]. It is important to note that here we studied LCRs generated only by this type of “hollow”, primary pacemaker cells, but we did not model LCRs in another type of (“peripheral”) sinoatrial node cells with RyR clusters also located inside the cells.

The dense clusters of RyRs are co-localized in the model with L-type calcium channels at 15 nm dyad junctions between JSR and the sarcolemma forming couplons (or Ca release units). Each couplon has one JSR compartment containing calsequestrin. The JSR receives Ca from the adjacent free SR through a diffusion resistance representing the several fine, tubular nexi. Ca from the JSR is released into the dyadic space through open RyRs at a rate proportional to the free Ca gradient between the JSR and the dyadic space at the location of each RyR. The dyadic space of each couplon is discretized into a two-dimensional grid of 10 nm squares on which local calcium and diffusible buffer evolve according to reaction-diffusion equations, which are integrated along with the calcium concentration in the JSR compartment. RyRs are located at 30 nm spacing, and L-type channels randomly placed. Ca diffuses from the edges of the cleft into the adjacent cytosol. Joint gating of RyRs and L-type channels is simulated by a custom-modified Gillespie Monte Carlo algorithm that generates an exact realization of the high dimensional, variable-rate Markov process controlled by voltage and instantaneous local calcium [29].

### Programming and data analysis

In cardiac pacemaker cells the critical region for the clocks coupling is under cell membrane. It is the local Ca under the cell membrane that activates Na/Ca exchanger accelerating diastolic depolarization. Thus, while the model predicts Ca dynamics within the entire cell, here we examined Ca only under the cell membrane. Since the cell shape is approximated by a toroid, all examined signals under the membrane are represented by a 2D array of Ca intensities in respective sub-membrane voxels with a size of 100 x 100 x 100 nm (Fig 1). Examples of our model simulations of Ca dynamics (near the cell membrane and in cross sections) along with action potentials and NCX current at different Pup are given in S1 and S2 Movies.

Here we developed new, unique computer algorithms for automatic detection and classification of LCRs. The LCRs were detected in series of consecutive images of simulated Ca dynamics within 100 nm under the cell membrane. The computer algorithms were implemented in C++ within Microsoft Visual Studio 2010. Our original C++ source code of the algorithms is available at https://www.nia.nih.gov/research/labs/lcs/complex-lcr-detector. For visualizations we coded with OpenGL also in Microsoft Visual Studio 2010. Rather than having a single cut off amplitude to find Ca sparks [14], our new algorithm is capable of also differentiating between high amplitude and low amplitude neighboring release events. In other words, the new algorithm detects peaks of a wide range of amplitudes. The algorithm also tracks the LCR complex spatiotemporal evolution, including births, deaths, separations, and collisions. It is important to note that while the time tick of the model simulations was 0.05 ms, the simulation output (and our analysis) was performed with a time interval of 5 ms that satisfactory describes the LCR dynamics. This simplification substantially accelerated data output, storing, and computation. We analyzed the simulations within the time window starting at MDP and ending at -50 mV (at the threshold of L-type channel activation and before the beginning of action potential-induced Ca transient).

## Supporting information

S1 Movie Simulations of local Ca in submembrane cytosol layer and two cross-section (along and across the cell) along with action potentials (red, V_m_ in mV), NCX current (green, I_NCX_ in pA) and average Ca in submembrane layer of cytoplasm (blue, Ca_subCyt_, in 0.1 μM per unit scale). SR Ca pumping rate Pup was 4 mM/s. The local Ca signal amplitudes are coded by red shades starting with 0.1 μM (pure black) and reaching saturation (pure red) at 5 μM. Light-blue dots illustrate JSR refilling with Ca.
(MP4)

S2 Movie Numerical model simulations as in S2 Movie but at a much higher SR Ca pumping rate Pup of 24 mM/s. (MP4)

S3 Movie Automatic detection of LCRs in submembrane cytosol layer during a part of the diastolic period from MDP to -50 mV, before activation of I_CaL_. LCR areas are filled by red color. Simple LCRs are shown with blue border. Other border colors were used to show complex LCRs. SR Ca pumping rate Pup was 4 mM/s.
(WMV)

S4 Movie Automatic detection of LCRs with the same algorithm as in S3 Movie but at a much higher SR Ca pumping rate Pup of 24 mM/s (WMV)

S5 Movie Automatic detection of LCRs with the same algorithm as in S3 and S4 Movies but at an intermediate (moderate) SR Ca pumping rate Pup of 12 mM/s that was assigned to a basal state action potential firing in prior numerical studies. (WMV)
